# Current use of D-dimer for the exclusion of venous thrombosis in hospitalized patients

**DOI:** 10.1038/s41598-022-16515-6

**Published:** 2022-07-20

**Authors:** Nitzan Karny-Epstein, Ran Abuhasira, Alon Grossman

**Affiliations:** 1 Internal Medicine B, Rabin Medical Center, Beilinson Campus, Jabotinsky 39 St., Petah-Tikva, Israel; 2 Sackler Faculty of Medicine, Tel Aviv University, Tel Aviv, Israel

**Keywords:** Geriatrics, Medical imaging, Biomarkers, Risk factors, Diseases, Haematological diseases

## Abstract

D-dimer assay’s utility for excluding venous thromboembolism (VTE) in hospitalized patients is debatable. We aimed to assess the current use of D-dimer as a diagnostic tool for excluding VTE in hospitalized patients and examine a mandatory age-adjusted D-dimer (AADD) threshold for diagnostic imaging. Retrospective cohort study between 2014 to 2019 that included patients from medical and surgical wards with a positive AADD result drawn during their hospitalization. The outcomes were determining a D-dimer threshold requiring further evaluation and assessing the prognostic value of D-dimer in predicting clinically relevant VTE in hospitalized patients. The cohort included 354 patients, 56% of them underwent definitive diagnostic imaging, and 7.6% were diagnosed with VTE after a positive AADD within 90 days of follow-up. Mortality rates were higher in patients diagnosed with VTE (33.3% vs. 15.9%, p = 0.03). Patients with pneumonia and other infectious etiologies were less likely to be further evaluated by definitive imaging (p = 0.001). Patients with a respiratory complaint (p = 0.02), chest pain (p < 0.001), or leg swelling (p = 0.01) were more likely to undergo diagnostic imaging. Patients with D-dimer levels > X2 the AADD were at increased risk of VTE [OR 3.87 (1.45–10.27)]. At 90 days of follow-up, no excess mortality was observed for patients without diagnostic evaluation following elevated AADD. D-dimer may be used in hospitalized patients to exclude VTE using the traditional AADD thresholds, with a high negative predictive value. D-dimer levels > X2 the AADD usually mandates further diagnostic imaging, while lower levels, probably do not require additional workup, with a sensitivity of almost 80% and no excess mortality.

## Introduction

Venous thromboembolism (VTE) is a significant complication of hospitalization due to its diagnostic challenge, requiring a high level of suspicion, and increased morbidity and mortality rates^1,2^. Moreover, the diagnosis usually requires the performance of CTPA (computed tomography pulmonary angiography), exposing this susceptible population to contrast allergic reactions and nephrotoxicity, radiation, and its implications^3^, as well as being both time-consuming and expensive^4^.

D-dimer assay, along with clinical decision rules (CDRs), such as the Well’s criteria^5^ and revised Geneva score^6^, are well-validated and reliable methods for excluding VTE in out-patients and emergency-room visitors^7^.

D-dimer levels increase with age and in hospitalized patients due to significant co-morbidities and acute care settings^5,8^. As a result, information regarding the ability to rule out VTE based on a D-dimer assay, with or without CDRs, in hospitalized patients is scarce^9,10^. The use of age-adjusted D-dimer (AADD) was well-validated in several studies in the recent decade^11–13^, and it is used in combination with varied risk assessment scores for pulmonary embolism (PE)^14^.

Two systematic reviews and meta-analyses evaluated CDRs for diagnosing pulmonary embolism in hospitalized patients using the Wells criteria with the D-dimer test concluded that their use as diagnostic tools in hospitalized patients is safe to rule out PE. Still, only a small percentage of patients can be excluded from further imaging studies^15^, even when the age-adjusted D-dimer threshold is applied^16^. The negative predictive value of the D-dimer assay as a single tool to rule out VTE in hospitalized patients was demonstrated in some studies with varied results^7,17–19^. As a result, there has been an increase in diagnostic imaging and over-diagnosis of VTE by imaging^20^, with many incidental findings of low clinical significance requiring further evaluation^21^.

In this study, we used real-world experience in a tertiary care hospital to evaluate the D-dimer diagnostic threshold in the inpatient population and its implication on patients' subgroups for further evaluation and diagnosis of VTE and mortality rates.

## Methods

### Study design, population, and outcomes

This is a retrospective cohort study in a tertiary care hospital in central Israel. The study included adult hospitalized patients between January 2014 and December 2019 who had a positive AADD (D-dimer HS 500, automated latex immunoassay, HemosIL^®^) result during hospitalization in general and surgical wards. Exclusion criteria were tests taken before admission or after imaging studies were performed to eliminate patients examined for other reasons than VTE diagnosis and hospitalization in obstetrics and gynecology departments. Only the first value was used in cases of more than one admission with a D-dimer test throughout the study period.

The study objectives were assessment of the association between D-dimer and clinically relevant VTE in hospitalized patients; determination of a D-dimer threshold requiring further evaluation in patients above 50 years of age with a positive AADD, and identification of patient subgroups with a positive AADD assay that can safely be excluded from further definitive testing.

### Clinical data extraction and definitions

All hospitalizations with at least one D-dimer test were extracted from the hospital's computerized medical record. Only the first value was used in the case of several D-dimer tests for the same patient. Each medical record was reviewed manually by a physician (NKE), and the following information was extracted: age at admission, sex, smoking status, specific ward admitted, history of VTE, chronic illnesses (congestive heart failure; chronic obstructive pulmonary disease (COPD); obesity; active malignancy; stroke, atrial fibrillation, functional status; surgery/procedure in the 30 days before admission; etiology of admission was further subcategorized (see Supplementary Table 1). Additional data were collected, including symptoms at admission or during hospitalization (chest pain, dyspnea, asymmetric leg swelling/change in one leg circumference, hemoptysis); signs: hypoxia (oxygen saturation below 95%), hypotension (below 90 mmHg systolic or 60 mmHg diastolic), tachycardia (above 100 bpm, beats per minute); hospital length of stay, day of D-dimer test, anticoagulation treatment prior and during the hospital stay and anticoagulation prevention according to Padua score^22^ during hospitalization, use of central catheters. Diagnostic imaging was completed during admission and through 3 months of follow-up, diagnosis of VTE during hospitalization, and 3 months following discharge. All-cause mortality at admission and up to 3 months following discharge.

A positive D-dimer test was defined as above 500 ng/mL for patients under 50 years. For patients above 50 years of age, we defined the AADD cutoff as age × 10. Definitive diagnostic tests for VTE were defined as ultrasound Doppler to diagnose deep vein thrombosis, CTPA, perfusion ventilation lung scan, and cardiac computed tomography, which was reported as beneficial for the diagnosis of VTE by others^23,24^.

We also obtained data on patients with a negative AADD that were further evaluated with imaging studies.

### Statistical analysis

The results are presented by means ± SDs for continuous variables, medians and interquartile ranges for ordinal variables, and percentages for categorical data. When appropriate, we made univariate comparisons using the χ^2^-test for categorical variables and the Student's *t* test or Mann–Whitney test for quantitative variables.

Since the AADD defines only the cutoff from which a D-dimer test is considered positive, but the D-dimer values remain identical for all ages, we created a variable by dividing the D-dimer value in the AADD cutoff for each patient above the age of 50. For patients under the age of 50, we divided the D-dimer value by 500.

We used multivariable logistic regression to assess the factors associated with a diagnosis of VTE during the hospitalization or within 3 months of discharge. Variables with a significance of < 0.1 in the univariate analysis were inserted into the multivariable model, which included the following variables: age above 65 years, sex, D-dimer value divided by the age-appropriate cutoff, being immobilized prior to the hospitalization, a diagnosis of diabetes mellitus and the Padua score on admission. We used receiver operating characteristic (ROC) curve analysis to evaluate the accuracy of the D-dimer value/age-adjusted upper normal limit in detecting VTE. The area under the ROC curve (AUC) was used to summarize diagnostic accuracy, with 1.0 representing perfect discrimination and 0.5 representing chance discrimination.

A p-value of 0.05 or less (two-sided) was considered statistically significant. Statistical analyses were performed using SPSS version 25.0 (IBM Corp., Armonk, NY, USA).

### Ethics

This study was approved by the Rabin Medical Center institutional review board (IRB) Committee (confirmation number 0517-20). All clinical investigations were conducted according to the principles expressed in the Declaration of Helsinki. The IRB approval exempted the study from informed consent due to the retrospective data collection nature.

## Results

**Figure 1:**
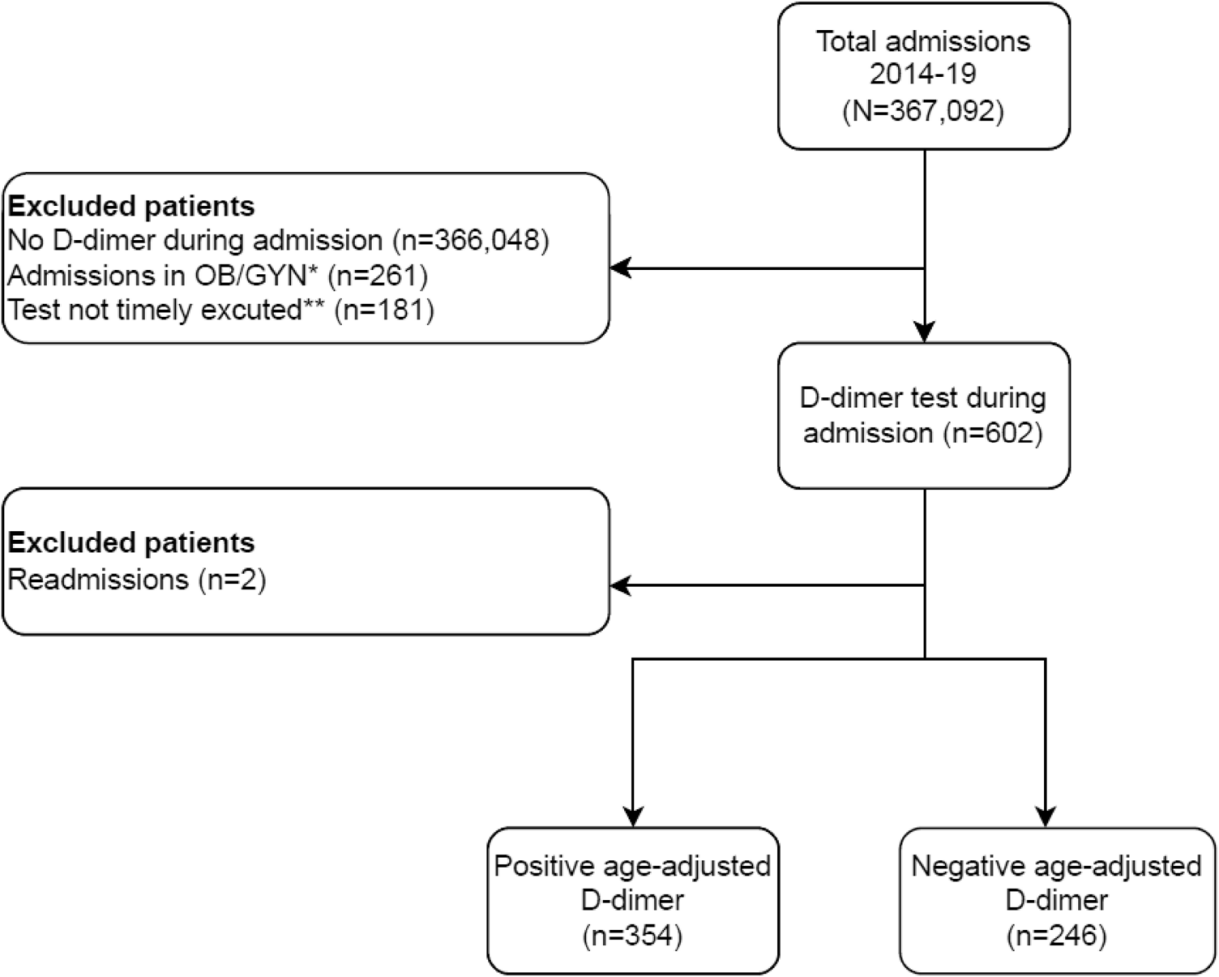
Flow diagram of the study cohort. *OB/GYN obstetrics and gynecology admissions during pregnancy and post-partum period. **D-dimer test drawn before admission or after definitive imaging executed.

Between January 2014 and December 2019, 367,092 admissions were identified in our institution. For 1044 admissions, a D-dimer assay was used during the hospitalization. Two hundred sixty-one admissions were excluded due to pregnancy or postpartum status, and 181 admissions were excluded for tests executed in the emergency department before admission or examination drawn after imaging, as depicted in Fig. 1.

A total of 354 admissions with a positive AADD assay during the hospitalization were used as the study cohort, 95.5% of the patients from medical wards and 4.5% from surgical wards. The cohort was composed of patients aged 18–96 years, with a median age of 69 years, 55% female (Table 1), and the median value of D-dimer was 1498 ng/mL. A total of 27 cases (7.6%) were diagnosed with VTE after a positive D-dimer test.

**Table 1:** Baseline characteristics of all hospitalized patients with a positive age-adjusted D-dimer test. Significant values are in bold.

Variable	All patients (N = 354)	No VTE (n = 327)*	VTE (n = 27)*	P-value (No VTE vs. VTE)
Female, n (%)	198 (55.93)	180 (55.05)	18 (66.67)	0.24
Age (years), median (IQR)	69 (57–79.5)	69 (56.8–79)	77 (66–84)	0.07
First D-dimer value (ng/mL), median (IQR)	1498 (1036–2947.5)	1431 (1017.3–2440)	5118 (1642–18,700)	**< 0.001**
D-dimer admission day, median (IQR)	2 (2–4)	2.5 (2–4)	2 (2–4)	0.06
Padua score, median (IQR)	3 (1–5)	3 (1–5)	3 (2–6)	0.38
In-hospital death, n (%)	31 (8.76)	28 (8.56)	3 (11.11)	0.65
Death in-hospital or within 3 months of discharge, n (%)	61 (17.2)	52 (15.9)	9 (33.3)	**0.03**
**Anticoagulation**				
Chronic anticoagulation (prior to admission), n (%)	17 (4.8)	15 (4.59)	2 (7.41)	0.51
Anticoagulation on admission, n (%)	211 (59.6)	188 (57.49)	23 (85.19)	**0.005**
Prophylactic anticoagulation dosing (of patients anticoagulated), n (%)	119 (56.4)	116 (61.7)	3 (13.0)	**< 0.001**
**Symptoms during the hospitalization**				
Chest pain, n (%)	108 (30.51)	98 (29.97)	10 (37.04)	0.44
Dyspnea, n (%)	215 (60.73)	198 (60.55)	17 (62.96)	0.81
Asymmetric leg swelling, n (%)	64 (18.08)	59 (18.04)	5 (18.52)	0.95
Cough, n (%)	32 (9.04)	30 (9.17)	2 (7.41)	0.76
Hemoptysis, n (%)	8 (2.26)	8 (2.45)	0 (0)	0.41
**Clinical condition**				
Heart rate > 100 beats per minute, n (%)	145 (40.96)	135 (41.28)	10 (37.04)	0.67
Oxygen saturation < 95%, n (%)	221 (62.43)	202 (61.77)	19 (70.37)	0.38
Blood pressure under 90/60 mmHg, n (%)	218 (61.58)	199 (60.86)	19 (70.37)	0.33
**Diagnoses**				
DIC during the hospitalization, n (%)	28 (7.91)	25 (7.65)	3 (11.11)	0.52
Pneumonia during the hospitalization, n (%)	72 (20.34)	69 (21.1)	3 (11.11)	0.22
Dementia during the hospitalization, n (%)	11 (3.11)	11 (3.36)	0 (0)	0.33
PICC line during the hospitalization, n (%)	13 (3.67)	13 (3.98)	0 (0)	0.29
Procedure 30 days before the hospitalization, n (%)	17 (4.83)	16 (4.92)	1 (3.7)	0.78
Surgery 30 days before the hospitalization, n (%)	12 (3.4)	12 (3.68)	0 (0)	0.31
Hormonal therapy, n (%)	12 (3.4)	12 (3.68)	0 (0)	0.31
Personal history of VTE, n (%)	24 (6.78)	21 (6.42)	3 (11.11)	0.35
**Functional status before hospitalization**				
Independent, n (%)	246 (71.30)	231 (72.64)	15 (55.56)	0.10
Mentally frail, n (%)	57 (16.52)	52 (16.35)	5 (18.52)	0.72
Immobilization, n (%)	42 (12.17)	35 (11.01)	7 (25.93)	**0.02**
**Comorbidities**				
Smoking, n (%)	117 (33.05)	109 (33.33)	8 (29.63)	0.69
Chronic obstructive pulmonary disease, n (%)	79 (22.32)	73 (22.32)	6 (22.22)	0.99
Heart failure, n (%)	47 (13.28)	44 (13.46)	3 (11.11)	0.73
Obesity, n (%)	68 (19.21)	64 (19.57)	4 (14.81)	0.55
Diabetes mellitus, n (%)	108 (30.51)	104 (31.8)	4 (14.81)	0.07
Hypertension, n (%)	187 (52.82)	174 (53.21)	13 (48.15)	0.61
Stroke, n (%)	36 (10.17)	33 (10.09)	3 (11.11)	0.87
Malignancy, n (%)	82 (23.16)	77 (23.55)	5 (18.52)	0.55
Atrial fibrillation, n (%)	16 (4.52)	14 (4.28)	2 (7.41)	0.45
**Primary admission etiology**				
Cardiac, n (%)	52 (14.69)	49 (14.98)	3 (11.11)	0.59
Electrolyte abnormality, n (%)	8 (2.26)	8 (2.45)	0 (0)	0.41
Hematologic, n (%)	12 (3.39)	11 (3.36)	1 (3.7)	0.93
Hepatobiliary, n (%)	15 (4.24)	15 (4.59)	0 (0)	0.26
Infectious, n (%)	100 (28.25)	93 (28.44)	7 (25.93)	0.78
Neurological, n (%)	24 (6.78)	22 (6.73)	2 (7.41)	0.89
Pain control, n (%)	6 (1.69)	5 (1.53)	1 (3.7)	0.40
Respiratory, n (%)	87 (24.58)	77 (23.55)	10 (37.04)	0.12
Surgical, n (%)	27 (7.63)	26 (7.95)	1 (3.7)	0.42
Syncope, n (%)	23 (6.5)	21 (6.42)	2 (7.41)	0.84

*VTE and "No VTE" are defined as venous thromboembolism definitively diagnosed during the hospital admission or within 3 months of discharge.

*VTE* venous thromboembolism, *DIC* disseminated intravascular coagulopathy, *PICC* peripherally inserted central catheter.

Patients with a diagnosis of VTE had significantly higher D-dimer levels (median 5118 ng/mL vs. 1431, p < 0.001) and were more likely to be immobilized (25.93% vs. 11.01%, p = 0.02). Patients with VTE diagnosis were treated more with therapeutic dosing of anticoagulation (during hospitalization (85.2% vs. 57.5%, p = 0.005), while prophylactic anticoagulation was common in the non-VTE group (61.7% vs. 13%, p < 0.001). No significant difference was found in age, Padua score, chronic use of anticoagulants before admission, symptoms, signs, and co-morbidities.

All-cause in-hospital mortality and 3-months following hospital discharge were higher in the VTE group compared to the non-VTE group (33.3% vs. 15.9%, p = 0.03).

Table 2 compares patients with and without definitive diagnostic imaging for VTE. Of the entire cohort, 56% were further evaluated for the presence of VTE by a definitive imaging test, in most cases (78%) a CTPA. The group's age, sex, and 3-months mortality following hospital discharge were similar. A small but statistically significant difference was found for in-hospital mortality (12.3% vs. 6.0%, p = 0.04). Further investigation of in-hospital mortality in patients without a definitive diagnostic test is shown in Supplementary Table 2. Most of these patients died from advanced diseases associated with extremely high D-dimer levels, such as septic shock, metastatic malignancies, disseminated intravascular coagulation, and liver failure. Patients with prophylactic anticoagulation treatment were less likely to undergo further evaluation (81.4% vs. 39%, p < 0.001).

**Table 2:** Baseline characteristics comparing patients with a positive age-adjusted D-dimer test and imaging evaluations. Significant values are in bold.

Variable	No definitive test (n = 155)*	Definitive test (n = 199)*	P-value
Female, n (%)	78 (50.32)	120 (60.3)	0.06
Age (years), median (IQR)	69 (58.8–79.3)	69 (57–80)	0.96
First D-dimer value (ng/mL), median (IQR)	1411 (1002.3–2439.3)	1584 (1060–3182)	0.2
D-dimer admission day, median (IQR)	3 (2–5)	2 (2–4)	**< 0.001**
Padua score, median (IQR)	4 (2–5)	2 (1–5)	**0.002**
In-hospital death, n (%)	19 (12.26)	12 (6.03)	**0.04**
In-hospital death or within 3 months of discharge, n (%)	32 (20.6)	29 (14.6)	0.13
**Anticoagulation**			
Chronic anticoagulation (prior to admission), n (%)	9 (5.81)	8 (4.02)	0.44
Anticoagulation on admission, n (%)	86 (55.48)	125 (62.81)	0.16
Prophylactic anticoagulation dosing (of patients anticoagulated), n (%)	70 (81.4)	49 (39.2)	**< 0.001**
**Symptoms during the hospitalization**			
Chest pain, n (%)	27 (17.42)	81 (40.7)	**< 0.001**
Dyspnea, n (%)	88 (56.77)	127 (63.82)	0.18
Asymmetric leg swelling, n (%)	19 (12.26)	45 (22.61)	**0.01**
Cough, n (%)	16 (10.32)	16 (8.04)	0.46
Hemoptysis, n (%)	3 (1.94)	5 (2.51)	0.72
**Clinical condition**			
Heart rate > 100 beats per minute, n (%)	75 (48.39)	70 (35.18)	**0.01**
Oxygen saturation < 95%, n (%)	97 (62.58)	124 (62.31)	0.96
Blood pressure under 90/60 mmHg, n (%)	102 (65.81)	116 (58.29)	0.15
**Diagnoses**			
DIC during the hospitalization, n (%)	18 (11.61)	10 (5.03)	**0.02**
Pneumonia during the hospitalization, n (%)	44 (28.39)	28 (14.07)	**0.001**
Dementia during the hospitalization, n (%)	4 (2.58)	7 (3.52)	0.61
PICC line during the hospitalization, n (%)	8 (5.16)	5 (2.51)	0.19
Procedure 30 days before the hospitalization, n (%)	9 (5.81)	8 (4.06)	0.45
Surgery 30 days before the hospitalization, n (%)	4 (2.6)	8 (4.02)	0.46
Hormonal therapy, n (%)	3 (1.94)	9 (4.55)	0.18
Personal history of VTE, n (%)	14 (9.03)	10 (5.03)	0.14
**Functional status before hospitalization**			
Independent, n (%)	94 (61.84)	152 (78.76)	**0.001**
Mentally frail, n (%)	35 (23.03)	22 (11.4)	**0.003**
Immobilization, n (%)	23 (15.13)	19 (9.84)	0.13
**Comorbidities**			
Smoking, n (%)	50 (32.26)	67 (33.67)	0.78
Chronic obstructive pulmonary disease, n (%)	38 (24.52)	41 (20.6)	0.38
Heart failure, n (%)	19 (12.26)	28 (14.07)	0.62
Obesity, n (%)	28 (18.06)	40 (20.1)	0.63
Diabetes mellitus, n (%)	50 (32.26)	58 (29.15)	0.53
Hypertension, n (%)	82 (52.9)	105 (52.76)	0.98
Stroke, n (%)	18 (11.61)	18 (9.05)	0.43
Malignancy, n (%)	35 (22.58)	47 (23.62)	0.82
Atrial fibrillation, n (%)	5 (3.23)	11 (5.53)	0.30
**Type of test used†**			
Chest CT, n (%)	–	153 (76.88)	–
A duplex ultrasound, n (%)	–	62 (31.16)	–
V/Q scan, n (%)	–	16 (8.04)	–
**Primary admission etiology**			
Cardiac, n (%)	20 (12.9)	32 (16.08)	0.40
Syncope, n (%)	6 (3.87)	17 (8.54)	0.08
Infectious, n (%)	58 (37.42)	42 (21.11)	**0.001**
Respiratory, n (%)	29 (18.71)	58 (29.15)	**0.02**
Pain control, n (%)	4 (2.58)	2 (1.01)	0.26
Surgical, n (%)	11 (7.1)	16 (8.04)	0.74
Hepatobiliary, n (%)	5 (3.23)	10 (5.03)	0.40
Electrolyte abnormality, n (%)	4 (2.58)	4 (2.01)	0.72
Hematologic, n (%)	8 (5.16)	4 (2.01)	0.10
Neurological, n (%)	10 (6.45)	14 (7.04)	0.83

*Positive test is defined according to age-adjusted D-dimer.

*Definitive test refers to a diagnostic test designed to diagnose venous thromboembolism – lower-extremity ultrasound with Doppler, ventilation-perfusion scan, or a computed tomography pulmonary angiography. This refers to tests during admission or within 3 months of discharge.

^†^Some patients did more than one definitive test.

*DIC* disseminated intravascular coagulopathy, *PICC* peripherally inserted central catheter.

D-dimer levels were drawn earlier through admission stay in patients with further evaluation (median day 2 vs. day 3, p < 0.001). Symptoms related to additional diagnostic imaging were chest pain and leg swelling (40.7% vs. 17.4%, p < 0.001, and 22.6% vs. 12.3%, p = 0.01, respectively). Patients with documented tachycardia were less likely to undergo diagnostic imaging (48.39% vs. 35.18%, p = 0.01). Patients classified as ambulatory were more likely to undergo imaging studies (78.76% vs. 61.84%, p = 0.001).

Patients diagnosed with pneumonia were less likely to undergo additional diagnostic imaging (22.6% vs. 12.39%, p = 0.005), as were patients with the diagnosis of disseminated intravascular coagulopathy on admission (11.61% vs. 5.03%, p = 0.02), and patients with any infectious etiology at admission (37.42% vs. 21.11%, p = 0.001), in contrast to patients with respiratory complaints who were more likely to undergo further imaging (29.15% vs. 18.71%, p = 0.02).

Multivariable analysis confirmed that age above 65 (OR 3.33, 95% CI 1.17–9.42), immobilization (OR 3.05, 95% CI 1.05–8.89), and an elevated D-dimer test above twice the AADD (OR 3.87, 95% CI 1.45–10.27) were all independent variables associated with a higher probability of the diagnosis of VTE. Diabetes mellitus was found to be a protective factor (Table 3).

**Table 3:** Multivariable logistic regression of venous thromboembolism during hospitalization or within 3 months of discharge.

Variable	Odds ratio (95% CI)
Age > 65 years	3.33 (1.17–9.42)
Male sex	0.61 (0.26–1.46)
D-dimer value/age-adjusted upper normal limit > 2	3.87 (1.45–10.27)
Functional status before hospitalization—bed-ridden	3.05 (1.05–8.89)
Diabetes mellitus	0.33 (0.11–0.99)
Padua score on admission	0.92 (0.76–1.12)

Sensitivity, specificity, negative and positive predictive values were calculated (Table 4) using additional data obtained on patients with a negative AADD and further imaging evaluation. The negative predictive value for VTE was 97.34% (CI 92.48–99.09%) in patients with a negative AADD.

**Table 4:** Sensitivity, specificity, positive and negative predictive values for the D-dimer test in the inpatient setting.

Statistic	Value (%)	95% CI
Sensitivity	90.00	73.47–97.80%
Specificity	30.14	24.14–36.68%
Positive predictive value	9.58	8.38–10.94%
Negative predictive value	97.34	92.48–99.09%
Accuracy	34.69	28.79–40.95%
Disease prevalence	7.6	

**Figure 2:**
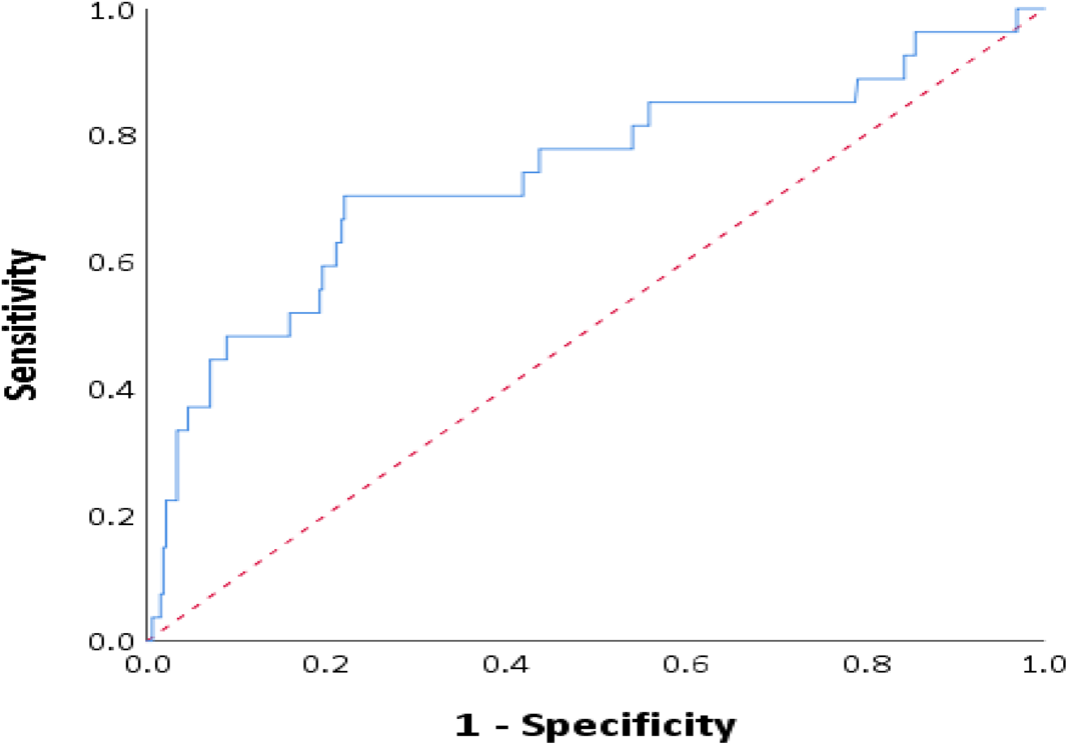
Receiver operating characteristic (ROC) analysis of diagnosis of venous thromboembolism by D-dimer value/age-adjusted upper normal limit ratio in hospitalized patients (AUC 0.74, 95% CI = 0.62–0.85). The Red dashed line represents the identity line (positive D-dimer values obtained during hospitalization; venous thromboembolism was diagnosed during the hospitalization or within 3 months of discharge).

Utilization of the D-dimer assay divided by the Age-adjusted upper normal limit is depicted in a ROC analysis for the prediction of VTE (Fig. 2); The AUC is 0.74, 95% CI = 0.62–0.85. A value of 2 in the D-dimer/AADD cutoff provides a sensitivity of 77.8% with a specificity of 48.9%, while a value of 4 provides a sensitivity of 70.4% with a specificity of 76.8%.

## Discussion

In this study, the utility of the D-dimer assay in hospitalized patients was evaluated for the diagnosis of clinically relevant VTE^25,26^. Our study was composed of patients hospitalized for various conditions in surgical and medical wards who were found to have a positive AADD. The management and outcomes of these patients were evaluated.

Patients diagnosed with VTE were more likely to be immobilized and with higher D-dimer serum levels. In-hospital and 90-day mortality combined were higher in the VTE group, as depicted in Table 1, whereas the in-hospital mortality rate alone was not significantly increased, probably due to a small number of patients and the cumulative morbidity of VTE later on^27^.

Mortality rates were similar in those who had a definitive imaging test and those who did not in the follow-up of 3 months. A slight but significant difference in in-hospital mortality was observed in patients with a positive AADD who did not undergo a definitive imaging test. When further assessing the causes of death in this group of patients, most did not die from VTE (Supplementary Table 2).

Patients who were more likely to be evaluated with imaging studies for a diagnosis of VTE had lower Padua scores^22^. Low Padua scores in this group could be explained by the absence of prophylactic anticoagulation therapy during hospitalization resulting consequently in a higher suspicion rate in this group, as demonstrated in Table 2.

Patients were more likely to undergo further imaging if they presented with chest pain or leg swelling, had no pneumonia or another infectious diagnosis upon admission, and were more ambulatory, consistent with former results that in the absence of another etiology for complaints, diagnosis of VTE is more likely, and should be further evaluated^16,28,29^.

Patients in this group were less tachycardic with a mean heart rate of 70 bpm compared to 75 bpm in the second group. We believe these results, although statistically significant, do not imply clinical use since the difference in the mean heart rate is negligible, although elevated heart rate (above 100 bpm) was found to be associated with VTE in hospitalized medical patients in the past^30^. On the contrary, patients with a respiratory etiology for admission (i.e., hypoxia, hemoptysis, pleural effusion, COPD and asthma exacerbation, pneumothorax, cough) were more likely to be evaluated with a definitive imaging test, similar to other reports^31^.

Patients defined on admission as mentally frail by the nursing staff were less likely to be evaluated by further imaging studies, perhaps due to the lack of informed consent needed for radio-contrast imaging. In a prospective study of hospitalized patients in a psychiatric hospital, VTE was high in catatonic and restrained patients and low in other patients not immobilized^32^.

It is interesting to note that D-dimer levels were similar between the groups with and without a definite test. It appears that physicians followed the notion that D-dimer levels during hospitalization are not very useful and used other clinical criteria to determine the need for a definitive diagnostic study. It could also be explained by using the D-dimer test for other utilities apart from VTE diagnosis or exclusion.

Moreover, the use of the D-dimer test was, in some cases, executed early through the hospital stay. This could imply that the cohort underrepresents the inpatient population and emphasizes presenting symptoms in admission. However, the mean day for the test was day 3, while the mean total hospital stay was 4 days.

In a multivariable regression analysis, age > 65, functional status, and high values of AADD > X2, the normal limit were independent risk factors for VTE during hospitalization and within 90 days of follow-up, in accordance with prior reports^19,27,29,30^. Sex and Padua score had no significant association^33,34^, while diabetes mellitus was a protective factor. Whether diabetes is a risk factor for VTE is a matter of debate, but the positive or negative association probably results from confounding by other factors and not diabetes itself^35^. This is perhaps the case in our results as well. Surprisingly, the Padua score was found to have no impact as a predictive value for VTE. This could be due to underscoring by the medical staff, as reported by others^36,37^, or by the low sensitivity of the Padua score for patients admitted to internal medical wards^34^.

We evaluated the negative predictive value of the D-dimer test in hospitalized patients and found similar results to that reported for ambulatory patients^8,38^. Due to a relatively small cohort and low prevalence, we chose to use the ROC curve, which allowed us to relate to sensitivity and specificity of the test independently to disease prevalence. According to the ROC curve, the discriminatory power of D-dimer divided by the upper limit AADD is 74% and is consistent with previously reported studies^19,39^. However, there is some controversy on this matter, Van der Hulle et al.^40^, in an earlier study, adjusted the Wells criteria for the elevation of D-dimer thresholds, which resulted in a smaller proportion of patients requiring further evaluation with CTPA. However, this reduction of "risk population" resulted in unacceptable diagnostic failure rates, as defined by the authors.

A recently published meta-analysis by Stals et al.^41^ examining the safety of CDRs and D-dimer assay showed that the failure rates in elderly patients and cancer-related pulmonary embolism previously reported have been overestimated due to misclassifications related to VTE and not to other co-morbidities.

Although based on the inpatient population alone, our results are aligned with the "YEAR" study^42^ in which, even in relatively high D-dimer levels, the assay is a reliable way to exclude PE in low to intermediate-risk patients.

Yet, there is still a debate since most patients in this group will have an intermediate to high risk for VTE, determined by CDRs and D-dimer levels^43,44^.

Our study examined a varied cohort from a tertiary care center. We found that among hospitalized patients with a positive AADD, using a cutoff of X2 the age-adjusted upper normal limit can identify almost 80% of clinically relevant VTE cases. Thus, the utility of the D-dimer test in hospitalized patients in ruling-out VTE with relatively low D-dimer levels and the presence of an alternative diagnosis is possible in terms of mortality rates and diagnosis of VTE during the hospitalization and in 3 months of follow up from hospital discharge.

Since this is a real-life observational retrospective study, we used clinically relevant VTE as primary endpoint in the evaluation of the D-dimer assay, this method was implemented and validated by others^25,26^. In a non-inferiority study on emergency visitors’ population Freund et al.^45^ showed that even when positive AADD is encountered up to 1000 ng/dL patients could be excluded from further evaluation, these findings are consistent with our results.

Further prospective evaluation of the AADD threshold in the inpatient population is required in order to implement the D-dimer assay in the evaluation of VTE in hospitalized patients.

### Study limitations

First, since this is a retrospective study, no data are available on asymptomatic VTE, a common issue of debate, and the ability to calculate an accurate Wells score is limited. Second, no information was available about the location of pulmonary emboli, which is important since sub-segmental PE is considered less clinically significant. Third, a definitive imaging test for VTE was available only for 60% of the patients with a positive AADD. Due to the nature of this study, risk stratification of patients into low, intermediate and high-risk groups was not optional. Moreover, since this is a retrospective study generalizability is also limited due to selection bias of patients with a D-dimer test taken during the hospitalization. It is imperative to mention that in our institute the use of the D-dimer test for hospitalized patients is limited since it is not well validated, hence it may also lead to a selection bias. We did not exclude patients prescribed anticoagulation therapy prior to admission, although this group was very small (less than 5% of the cohort). Nevertheless, patients’ medical records were studied for 3 months after the first suspicion of VTE, a method that has already been validated in previous studies^46^. The lack of difference in mortality rates implies that the outcome was not affected even if some VTE diagnoses were not identified. The strengths of this study are its relatively large cohort and the manual collection and validation of data instead of using codes of diagnoses.

## Conclusions

The study shows that the D-dimer test may be used in hospitalized patients for the exclusion of VTE. D-dimer levels > X2 the AADD usually mandates further diagnostic imaging, while lower levels, even those considered to be traditionally elevated may not require additional workup with a sensitivity of almost 80%. The study shows that when other etiologies than VTE exist, clinicians not infrequently select not to perform further diagnostic studies as suggested by VTE algorithms. No excess mortality in 90 days of follow-up was observed when implementing this rule.

Further studies are needed to evaluate prospectively the utility and proper threshold of the AADD.

## Supplementary Information

Supplementary Tables.

## Data Availability

The data used in the analysis of this study are not publicly available due to the requirements of the IRB Committee but are available from the corresponding author upon request.
