# Supplementary material for: Current use of D-dimer for the exclusion of venous thrombosis in hospitalized patients
Source: Sci Rep. 2022 Jul 20;12:12376. doi: 10.1038/s41598-022-16515-6 (PMC9300739; doi:10.1038/s41598-022-16515-6)
Supplement: Supplementary file 1 — Supplementary Tables. [file 41598_2022_16515_MOESM1_ESM.docx]

**Current use of D-dimer for the exclusion of venous thrombosis in hospitalized patients**

Nitzan Karny Epstein MD^a,b^, Ran Abuhasira MD PhD^a,b^, Alon Grossman MD MHA^a,b^

**Supplementary Tables**

**Table S1 - Primary admission etiologies**

| **Etiology Category** | **List of Diagnoses** |
| --- | --- |
| Cardiac | Chest pain, palpitations, atrial fibrillation and tachy-arrythmia, pericardial effusion and tamponade, aortic dissection, syncope. |
| Respiratory | hypoxia, hemoptysis, pleural effusion, chronic obstructive pulmonary disease and asthma exacerbation, pneumothorax, cough. |
| Neurologic | stroke, transient ischemic attack, diplopia, seizure, headache, dystonia, delirium. |
| Infectious | cellulitis, fever, fever and rash, sepsis, septic shock, fever of unknown origin. |
| Surgical | abdominal pain, upper and lower gastrointestinal bleeding, perianal abscess, small bowel obstruction, elective surgery, pancreatitis, hip fracture, orthopedic surgery. |
| Electrolyte abnormality | hypokalemia, hypercalcemia, hyponatremia, hypo and hyperglycemia, hyperosmolar hyperglycemic state, acute kidney injury. |
| Hematologic | anemia, pancytopenia, thrombocytopenia  hemolytic anemia, bone marrow transplant, acute myeloid leukemia, acute lymphoid leukemia. |
| Hepatobiliary | anasarca and leg edema, hepatic failure, hepatic encephalopathy. |
| Pain control | back pain, leg pain |

**Table S2 - Causes of death during hospitalization in patients with a positive age-adjusted D-dimer and no definitive imaging test (see Table 2).**

| **Cause of death** | **Number of patient (N=19)** |
| --- | --- |
| Severe pneumonia, severe septic shock, n (%) | 10 (52%) |
| Active metastatic malignancy to liver with disseminated intravascular coagulopathy, n (%) | 5 (26%) |
| Other metastatic malignancy complications (pleural effusion, liver failure), n (%) | 2 (10%) |
| Central nervous system vasculitis, n (%) | 1 (5%) |
| Advanced constrictive pericarditis, n (%) | 1 (5%) |

**Table S3 - Thresholds examined for AADD**

|  | **Sensitivity** | **Specificity** |
| --- | --- | --- |
| A cutoff point of 2 times the AADD | 82.8% | 34.5% |
| A cutoff point of 1.51 times the AADD | 89.7% | 20.2% |
| A Cutoff point of 3.95 times the AADD | 75.9% | 53.7% |
| A Cutoff point of D-dimer 1,261 ng/mL | 75.9% | 52.2% |
| A Cutoff point of D-dimer 513 ng/mL | 93.1% | 18.2% |
